# Complete Genome Sequences of a Mycobacterium smegmatis Laboratory Strain (MC^2^ 155) and Isoniazid-Resistant (4XR1/R2) Mutant Strains

**DOI:** 10.1128/genomeA.01520-14

**Published:** 2015-02-05

**Authors:** Abhilash Mohan, Jyothi Padiadpu, Priyanka Baloni, Nagasuma Chandra

**Affiliations:** a Department of Biochemistry, IISc, Bangalore, India; b Supercomputer Education and Research Centre, IISc, Bangalore, India; c Molecular Biophysics Unit, IISc, Bangalore, India

## Abstract

We report the whole genome sequences of a Mycobacterium smegmatis laboratory wild-type strain (MC^2^ 155) and mutants (4XR1, 4XR2) resistant to isoniazid. Compared to Mycobacterium smegmatis MC^2^ 155 (NC_008596), a widely used strain in laboratory experiments, the MC^2^ 155, 4XR1, and 4XR2 strains are 60, 128 and 93 bp longer, respectively.

## GENOME ANNOUNCEMENT

Emergence of drug resistance is a major bottleneck in tuberculosis treatment (1). Mycobacterium smegmatis MC^2^ 155, a nonpathogenic, fast-replicating mycobacterium (2), is widely used as a model system to study Mycobacterium tuberculosis. M. smegmatis MC^2^ 155 and M. tuberculosis share significant similarities in their genomes, including genes associated with mycolic-acid rich cell walls (3). Isoniazid (INH), a front-line clinical drug against M. tuberculosis, also inhibits M. smegmatis MC^2^ 155, albeit with a higher MIC. Here we report the whole-genome sequences of wild-type and drug-resistant strains of M. smegmatis MC^2^ 155.

The wild-type M. smegmatis MC^2^ 155 (MC^2^ 155) was subjected to increasing doses of INH, and INH-resistant strains (4XR1 and 4XR2) were selected using standard protocols (4, 5). Two colonies at 4-fold MIC were finally selected and sequenced along with MC^2^ 155^.^

The genomes of MC^2^ 155, 4XR1, and 4XR2 were sequenced using next-generation sequencing technology, at Genotypic Technology Private Limited, Bangalore, India, using the Illumina HiSeq platform. A total of 12,832,698, 13,636,408, and 13,636,408 unpaired reads of an average length of 101 bp for MC^2^ 155, 4XR1, and 4XR2, respectively, were obtained after trimming and clipping using Trimmomatic (v.0.30) (6). A template-based assembly of the genomes was performed using Bowtie2 (7). The MC^2^ 155 draft genome sequence was assembled using the M. smegmatis (NC_008596) strain as the template, while the 4XR1 and 4XR2 sequences were assembled by using the MC^2^ 155 strain. The draft genomes of MC^2^ 155, 4XR1, and 4XR2 were 6,988,269 bp (6,790 genes, 6,625 coding sequences [CDS] and 54 RNAs [47 tRNA, 6 rRNA, and 1 ncRNA]), 6,988,337 bp, and 6,988,302 bp (6,791 genes) long, respectively, with a GC content of 67.4%. The genomes were annotated using Rapid Annotation Transfer Tool (RATT) (8), with annotation from NC_008596 being transferred to MC^2^ 155 and the annotations of MC^2^ 155 transferred to 4XR1 and 4XR2.

The preprocessing of the assembled genome was performed using Picard tools (v1.119), while variant calling was performed using Genome Analysis Toolkit (GATK) (v3.1-1) (9). The GATK-prescribed best practices were followed to filter and accept single nucleotide polymorphisms (SNPs) and insertion and deletions (indels). The SNP and indel annotations for the strains were added using snpEff (10). A total of 133 indels and 90 SNPs were observed in MC^2^ 155. The 4XR1 strain showed 19 indels and 2 SNPs, while the 4XR2 strain showed 14 indels and 2 SNPs.

The genome sequences of these strains will be useful for obtaining a global understanding of the mechanism of drug resistance and also providing new insights into the biology of M. smegmatis, which can also be extrapolated to M. tuberculosis and may pave the way for exploring different treatment strategies.

### Nucleotide sequence accession numbers.

The draft genome sequences of MC^2^ 155, 4XR1, and 4XR2 have been deposited at DDBJ/EMBL/GenBank under the accession numbers CP009494 (strain MC2 155), CP009495 (strain INHR1), and CP009496 (strain INHR2).
